# LeptoDB: an integrated database of genomics and proteomics resource of *Leptospira*

**DOI:** 10.1093/database/bay057

**Published:** 2018-06-12

**Authors:** Shruti Beriwal, Nikhil Padhiyar, Deven Bhatt, Prabhakar D Pandit, Afzal Ansari, Kumari Snehkant Lata, Zuber M Saiyed, Vibhisha Vaghasia, Priyanka Sharma, Shivarudrappa B Bhairappanavar, Subhash Soni, Jayashankar Das

**Affiliations:** Gujarat Biotechnology Research Centre, Department of Science and Technology, Government of Gujarat, Gandhinagar, Gujarat 382011, India

## Abstract

Leptospirosis is a potentially fatal zoo-anthroponosis caused by pathogenic species of *Leptospira* belonging to the family of *Leptospiraceae*, with a worldwide distribution and effect, in terms of its burden and risk to human health. The ‘LeptoDB’ is a single window dedicated architecture (5 948 311 entries), modeled using heterogeneous data as a core resource for global *Leptospira* species. LeptoDB facilitates well-structured knowledge of genomics, proteomics and therapeutic aspects with more than 500 assemblies including 17 complete and 496 draft genomes encoding 1.7 million proteins for 23 *Leptospira* species with more than 250 serovars comprising pathogenic, intermediate and saprophytic strains. Also, it seeks to be a dynamic compendium for therapeutically essential components such as epitope, primers, CRISPR/Cas9 and putative drug targets. Integration of JBrowse provides elaborated locus centric description of sequence or contig. Jmol for structural visualization of protein structures, MUSCLE for interactive multiple sequence alignment annotation and analysis. The data on genomic islands will definitely provide an understanding of virulence and pathogenicity. Phylogenetics analysis integrated suggests the evolutionary division of strains. Easily accessible on a public web server, we anticipate wide use of this metadata on *Leptospira* for the development of potential therapeutics.

Database URL: http://leptonet.org.in

## Introduction

Leptospirosis is an emerging potentially fatal zoo-anthroponosis with a worldwide occurrence spanning developing as well as developed countries (1–3). This disease is caused by pathogenic *Leptospira* species belonging to family *Leptospiraceae*. The paramount importance of the disease is reflected because of the wide geographical distribution covering large spectrum of mammals, including both wild and domestic as reservoir host and humans serving as accidental hosts. Annually, 1.03 million cases are reported globally with ∼60 000 deaths with highest morbidity in the resource poor settings and where no routine surveillance is performed (4). Most notable outbreaks have occurred in countries like Nicaragua, Brazil and India, the USA (2) and South-East Asia Region countries (WHO South-East Asia Region report). Real time surveillance reports of 787 global alerts for Leptospirosis by healthmap (http://www.healthmap.org/en/) between 2007 and 2013 evidently suggested its epidemic potential (5).

Leptospires are thin, tightly coiled bacteria with high motility (2, 6) which can enter through cuts, abraded skin or mucous membranes such as conjunctival, oral or genital surfaces (7). It also spreads through direct or indirect contact with urine, soil, water or tissues of infected animals ensued by systemic dissemination. The systemic nature of the infection shows non-specific symptoms such as headache, fever, chills, muscle pain, vomiting and diarrhea. In addition, severe form named Weil’s syndrome has clinical manifestations including jaundice, meningitis, pulmonary hemorrhage, hepatic and renal dysfunction and cardiovascular collapse.

There are >250 serovars, which have been reported so far based on the structural heterogeneity in the carbohydrate component of their lipopolysaccharide (LPS) (8). Additionally, on the basis of DNA–DNA hybridization technique the genus is divided into 22 species. *Leptospira* species have relatively large genome between 3.9 and 4.6 Mbp with at least two circular replicons with an average guanine-cytosine (GC) content of 35–45% (9).

Leptospirosis mimics symptoms with other diseases like dengue, malaria and typhoid. So diagnosis based on symptoms is unreliable for specific identification. In addition, due to the ubiquitous nature of the disease, availability of accurate, effective and efficient methods for early detection is pre-requisite. Hence, laboratory diagnosis is vital to obtain conclusive results. Even after crossing a century of discovery of *Leptospira* and in the post-genomic era, the concept of universal leptospiral vaccine, still remains a long-term goal. No alternatives to classical immunization strategies are available till-date, which confers only short-term immunity restricted to serovars (with a need of booster doses) and severe side effects. Currently, bacterins are widely available for animals but only few countries allow their commercialization for human use (10). To overcome the drawbacks, effort needs to be focused on development of multi-epitope based cross-reactive vaccine.

In recent years, the frequency of leptospiral infection has steadily grown and non-availability of anti-leptospiral drug demands rigorous research. Although already available antibiotics like doxycycline, cephalosporins and penicillin are administered but no statistically significant evidence seems to be available that suggest the benefit of antibiotic therapy in the treatment of severe leptospirosis. Moreover, the course of drug development is multifaceted which may take several years for delivering specific anti-leptospiral drug. Thus, an integrated approach including *in silico* studies are beneficial in revealing potential drug and vaccine candidates.

Toward aiming for developing a universal diagnosis and vaccine candidate for this emerging disease and looking into the exponential contribution of global researchers, the need for development of a platform was realized which can be a complete package of multifaceted data with all necessary tools to visualize the same. Current release of our database (LeptoDB) provides information of 17 complete genomes and 496 draft genomes encoding 1.7 million proteins for 23 *Leptospira* species with >250 serovars comprising pathogenic, intermediate and saprophytic strains as types. We foresee this platform as an assistance to accelerate global research community working on this disease. Also, the detailed tutorial has been provided with stepwise instructions for the use of this system and underlying databases.

## Materials and methods

### Data collection and pre-processing

LeptoDB currently hosts 513 genome and proteome sequences covering 23 well-recognized *Leptospira* species including 17 complete and 496 draft genomes (contigs or scaffolds). Annotations of protein coding genes and genes coding for ribosomal ribonucleic acid (rRNAs), transfer ribonucleic acid (tRNAs) and non-coding RNA (ncRNA) are stored in LeptoDB.

### Molecular diagnostic primers

In the interest of thoroughness, literature was meticulously reviewed and mined for oligonucleotide primers, experimentally used for detection and diagnosis of *Leptospira*. A list of 151 primers was compiled with an average size range from 18 to 32 bp used till-date. All the information related to primers, i.e. their sequence, gene name, orientation and references of the respective primers from NCBI is available on the website. The compendium may be utilized for *Leptospira* detection and thus, profoundly exploitable for diagnosis during potential epidemics facilitating effective responses.

### Genomic islands

Genomic islands (GIs) are probable regions, which are horizontally transferred within bacteria, leading to microbial genomic adaptations. Their association with virulence-related genes may lead to pathogenic factor identification in the bacterial genomes (11). In a similar way, GI prediction and associated potential pathogenic target may be identified in *Leptospira* genome. GIs of 16 strains from five *Leptospira* species, of which complete genomes are available in NCBI, were predicted using IslandViewer4. It gives precise GI region boundaries and associated gene content by incorporating various methods like nucleotide bias and presence of mobility genes along with tRNA and tmRNA gene integration sites (IslandPath-DIMOB), codon usage bias (SIGI-HMM) and approach of comparative genomics (Island Pick) with highest precision of 91% (12). Resulting output gave information of GI island start and end, island associated genes and its functional product.

### Curation and compilation of epitope

The epitopes which were experimentally validated and exhibiting positive assays were extracted from IEDB database (13). Initially 39 epitopes were converged in LeptoDB. Each antigenic protein entry contains information regarding the epitope count, their B and T-cell assay and major histocompatibility complex (MHC)-ligand assay with an additional information such as epitope sequence, source molecule, start position, end position, tissue type, culture conditions etc. We shall keep updating the data on a rolling basis, conventionally integrating it for pathogenic species.

### Putative therapeutic drug targets

Prevention of *Leptospira* infection by controlling environmental factors is difficult to practice in developing countries and it is a challenge to develop safe and effective vaccine for >200 diverse serovars (1). Till-date no specific anti-leptospiral drug treatment for combating the severe infection is available. Moreover, the drug development is a multifaceted course, which may take several years due to diversified serovars distribution. To broadly understand, the mechanism of *Leptospira* infection and vital processes for its survival inside mammalian host and developing potential vaccines and drugs against pathogenic species of *Leptospira* promising targets needs to be identified. Cerqueira *et al*. also established that the *Leptospira interrogans* species is associated with severe human Leptospirosis, while the other strains like *Leptospira santarosai* have shown their association with pigs and cattle (14). Our initial effort focus on *L. interrogans*, which is the most frequently reported species (2) with *L. interrogans* serogroup Icterohaemorrhagiae representing more than 50% of the Leptospires encountered in human infections (15) and also has the largest corpus of scientific literature. Therefore, protein sequences of putative targets (16) of pathogenic *L. interrogans* serovars Copenhageni were subjected to *in silico* structure prediction. The molecular modeling of 38 proteins for determination of tertiary structures was performed. The template identification was done for each protein sequence by performing BLAST (17) search against PDB database (http://www.rcsb.org) (18) keeping the cut-off *E*-value of <0.01. The top hits for different proteins that were in the range from 30 to 100% identity were selected to build homology model using software MODELLER (19). The quality of the 3D models was evaluated using Verify 3D (20) and assessment was done using Rampage tool (21). The Ramachandran plot of the predicted models had more than ≥91% of the residues in the favorable region. All the modeled structures for *Leptospira* proteins provided in the resource can be visualized by Jmol (http://www.jmol.org/) (22) and can also be downloaded as PDB files. The List of PDB IDs, which were used as template to model *Leptospira* proteins are provided in the Supplementary Table S1. These protein structures may help in reckoning the binding of drugs to potential drug targets.

### CRISPR/Cas

All 16 *Leptospira* strains with assembly status of complete genome were considered for finding out the CRISPR/Cas systems. The chromosome and plasmid sequences from each strain were given as input for the online tool CRISPRone (http://omics.informatics.indiana.edu/CRISPRone) (23). All the sequences that were subjected to the prediction provided results as CRISPR/Cas genes and CRISPR repeats and spacer sequences.

### 16s RNA-based phylogeny analysis

In order to understand the close groups under pathogenic, saprophytic & intermediate nature of leptospirosis, in total 524 strains were included in the study for 16S rRNA analysis. All the 16s rRNA gene sequences were subjected to alignment using the ClustalW program (24) and phylogenetic tree using maximum likelihood method with 1000 bootstrap was constructed by MEGA7.0.20 (25). The improved visualization of the constructed phylogeny tree was prepared using EvolView (26). The phylogeny tree alignment is shown in Supplementary Figure S1.

## Database architecture and web interface implementation

**Figure 1.**
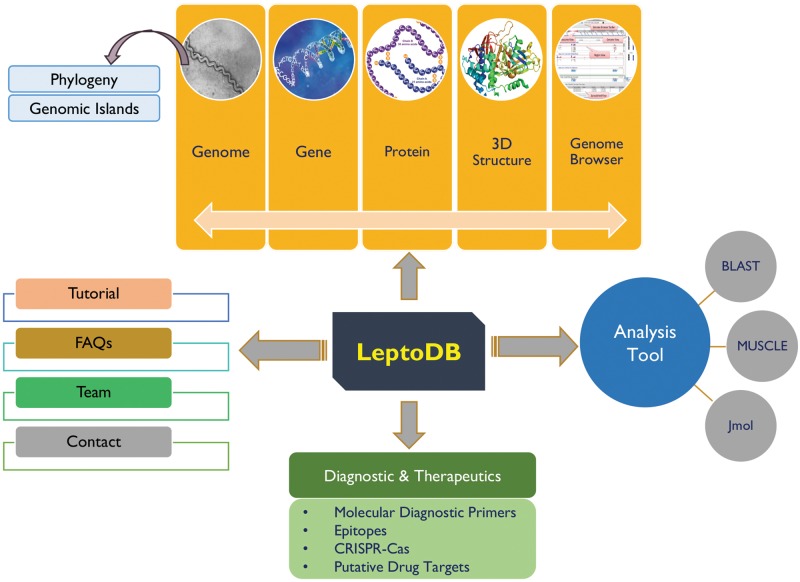
LeptoDB architecture.

LeptoDB has been developed as a comprehensive user-friendly data resource using Apache HTTP server 2.4.6 integrated with Python 2.7.5 and MySQL 5.7.18 on Linux server with CentOS 7 as operating system. PHP, JavaScript, HTML5 and CSS were used to develop the front-end of the database while MySQL was used to process the data at the back-end. The system has been designed using secure web-based application architecture of LeptoDB, which is displayed in Figure 1. Apache web server is dedicated to handle the requests from web clients and to interact with the back-end servers to serve the requests. Server-side operations are executed on a Linux server for creating complex pipelines of inputs and outputs for the necessary programs.

### Integration of web tools

In order to assist the users in searching, evaluating and retrieving data from LeptoDB, a user-friendly interface was developed.

### Incorporation of JBrowse

One of the important components of LeptoDB is integration of genomic and annotated genomic dataset on *Leptospira* species from published experiments for easy exploration and analysis for the users in JBrowse. JBrowse is an open source, mature and widely used application that is fast, intuitive and compatible with most browsers (27). It is highly flexible and customizable with the availability of plugin framework for analysis. JBrowse has been deployed to provide access to the emerging data from high-throughput sequencing technologies, which provides multiple zoom levels of resolution for base pairs in individual sequence reads across large genomic regions. The display of annotated sequence features allows seamless navigation between JBrowse and Locus summary for each feature. LeptoDB currently offers number of high-throughput DNA sequence data whether it is complete genome, contig or scaffold. Any new datasets available will be added to JBrowse manually.

### Analysis tools

LeptoDB facilitates very useful analysis and visualization tools to explore the genomic and proteomic data.

#### BLAST

The standard BLAST enables the users to search for sequence similarities exclusively against existing *Leptospira* genome sequences. We provide the *E*-value threshold 10 as default parameters and alignment format as pairwise alignment. Users can also choose different output format such as pairwise tabular format. The ‘Tabular’ output format will provide the details of percentage identity, alignment length, mismatch, gaps, percentage coverage, bit score and *E*-value.

#### Jmol

Intricate analysis and visualizations are indispensable for making sense of the complex 3D structures of protein to provide insight into their diverse functions in essential biological processes. To visualize the 3D structure of proteins, Jmol has been integrated which is a widely used open source Java script-based viewer. Jmol accepts 3D co-ordinate file (in PDB format).

#### MUSCLE (MUltiple Sequence Comparison by Log-Expectation)

MUSCLE: multiple sequence alignment visualization and manipulation tool has been provided to interactively edit and analyze the sequences (28).

## Results

To the best of our knowledge LeptoDB (freely accessible at www.leptonet.org.in) is a first attempt toward developing a comprehensive molecular resources covering wide range of genomic as well as proteomic information of *Leptospira* (Figure 1)*.* On clicking ‘Genomes’ we can view the *Leptospira* species currently available in LeptoDB. Further clicking on each species, the detailed information viz. strain, genome size in Mbp, GC %, number of genes and proteins are displayed. LeptoDB presently contains basic information for 1.96 million genes and 1.73 million proteins. Under the ‘Gene/Protein’ tab list, users get the list of all available strains of particular species and clickable links which provide information of gene, cds, tRNA, rRNA, ncRNA and protein. Each gene entry is presented with the information of locus tag, its genomic position and length or protein class. Similarly, all protein records cover information on sequence and amino acid count and accession number that has been cross-referenced with NCBI to provide an option of easy switching to the original source of information. Clicking on ‘InterProscan’ link provide details about protein family, domain, and biological process, molecular function and cellular component for each individual entry of protein.

The customized keyword-based search has been employed for searching and sorting the data for gene, protein and all RNAs. As the user will enter any keyword, the search engine will retrieve a list of functional classifications that contain the keyword entered by the user and display it to the user seamlessly. For example, user enters ‘hypothetical’ in search, they will be presented with the data which contains the entered keyword.

### Primer

Data of 151 primers with information of gene, species, primer name, primer sequence, orientation and technique with respective references cross-linked to NCBI may be accessed when clicked on the Primer tab.

### Genomic islands

Under the heading, ‘Genomic Islands’ the drop down list of 16 species is displayed. By selecting, the species from the drop down list of chromosome and plasmids are displayed. Clicking on the radio button will give the description of starting and end position of the genomic region in each chromosome where associated pathogenicity related genes are also displayed along with the strand information. The average number of genes among different strains in Chromosome I and Chromosome II ranges from 263 to 32, respectively, in total constituting ∼8% of the genome. No GIs were found in chromosome II of few pathogenic strains.

### Epitope

Clicking on the ‘Epitope’ tab the table of antigenic protein of pathogenic species of *Leptospira* is displayed with further the clickable links of presenting information on their T-cell assay (15), B-cell assay (84) and MHC ligand assay (6).

### 3D structure

Under the ‘3D structures’ list of therapeutic putative drug targets has been integrated for easy navigation with information of their respective sequences, Uniprot Id, gene symbol and 3D structure. Users can also download the structure file in PDB format. The 38 drug targets predicted comprises both cytoplasmic and membrane proteins, which are responsible for pathogenesis, antibiotic sensitivity and also enzymes responsible for intermediary metabolism etc.

### CRISPR/Cas

By clicking on a CRISPR tab, a table with information for 15 *Leptospira* strains with respective sequence id, and the Cas gene count with its type along with CRISPR repeat and spacer is exhibited. The major CRISPR/Cas system found among the pathogenic strains was type-I wherein three of them also had type-III. The *Leptospira biflexa* is a saprophytic species for which no CRISPR/cas system was detected. The total of 200 Cas genes with 187 repeats and 155 spacer sequences are represented for the above mentioned strains.

### Analysis tools

#### JBrowse

**Figure 2.**
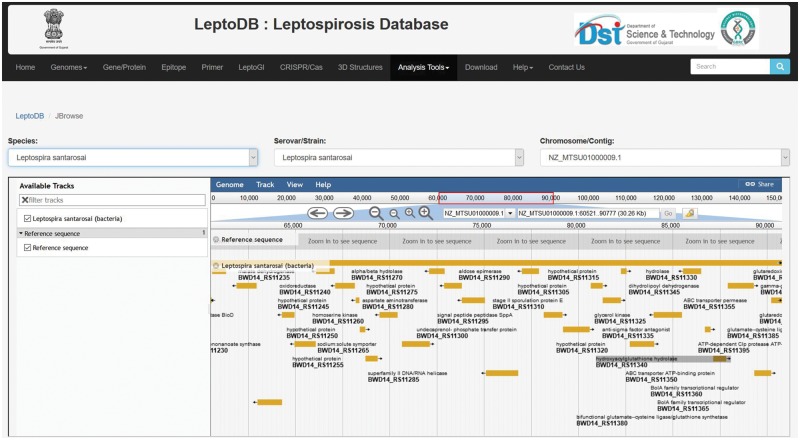
JBrowse showing annotated gene features.

All genome sequences in spite of their assembly status complete or draft (contig or scaffold) are mapped and can be visualized and interpreted through JBrowse framework (Figure 2). The user can select the species from drop down list and then select serovars followed by chromosome/contig, which opens up the JBrowse interface where user can view reference sequence of the genome with respect to its serovars and chromosome. The track provides list of locus tags for each gene and each locus is clickable giving details including gene name, symbol, identifiers, location, length, sequence and product. So far, Genome Browser is integrated to view a sequence feature (e.g. a gene) of interest in the context of its surrounding region and other features. User can easily upload their data files to JBrowse or paste URLs, where data is present to visualize and compare different tracks and hence analyze if there are similarities/dissimilarities between tracks. Additionally, custom tracks also enables comparison of genome tracks of different closely related organisms to study the variations in genome architecture across the length of the genome.

#### Jmol

**Figure 3.**
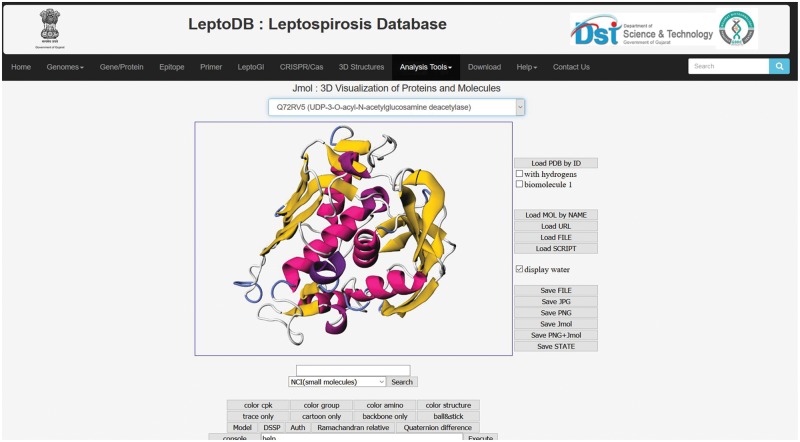
3D structure visualization in Jmol.

The integrated Jmol, 3D structural viewer will allow users to view the 3D structure of 38 putative drug targets which were generated as a part of the LeptoDB project (Figure 3).

#### BLAST

BLAST permits the user to perform a search or compare a query sequence against all *Leptospira* genomes in the database and result is generated in both text and tabular format.

#### MUSCLE

MUSCLE creates alignments of user-defined sequences with average accuracy comparable with or superior to the best available methods for fast editing and viewing of multiple sequence alignment.

#### Download

Users can download all the genome and proteome sequences and annotations available in LeptoDB through ‘Download’ page.

#### 16s RNA-based phylogeny analysis

The phylogenetic tree based on 16s RNA gene sequences of 524 serovars provides a bird’s eye view of *Leptospira* taxonomy and evolution which clearly suggests that 16s RNA gene is effective in classifying the strains at the clade level. Also, the analysis made a clear group among 497 pathogenic 9 intermediate and 15 saprophytic serovars. The phylogeny tree alignment is provided in the Supplementary Figure S1.

## Discussion

Leptospirosis is an emerging disease due to the diversity of *Leptospira* strains and its pathogenicity therefore, it requires significant attention of the researchers community for the development of effective diagnostics and therapeutics. As genome sequencing continues at its inexorably rapid pace, the increasing number of sequenced Genomes and several pathogenic serovars and multitude of animal species, it infects, it becomes apparent to study different aspects of *Leptospira* spp. including its evolution, diversity, genetics, pathogenicity and its biology. While availability of PubMLST database allows the users to query their allele sequence and sequence types along with the knowledge of the epidemiological data of the isolate, still abundant information needs to be deciphered (29). Therefore, there is a growing need for a unified platform, which integrates curated data along with the necessary analysis tools. A methodical approach is applied for building LeptoDB wherein wide range of information, interesting findings and analysis has been amalgamated. Indeed, the wide availability of Next generation sequencing (NGS) technology has prompted thorough investigation of genome data and improved understanding of pathogenesis of *Leptospira.* Studying whole genome and comparative biology seems imperative provided the breadth of pathogenic serovars and animals infected. As on 5 March 2018, genomic data of 513 *Leptospira* serovars with distribution of pathogenic (94%), saprophytic (3.5%) and intermediate (2.5%) implies data on pathogenic strains dominates the database proposing the emergence of genome level studies to evade and manage the disease. Previously reported numerous genomic studies illuminate our understanding of molecular mechanisms and pathogenesis. Seminal work done by Nascimento *et al*. on genome sequences of highly pathogenic species *L. interrogans* serovars revealed differences in genes involved in biosynthesis of LPS O side chains, in adhesins and significant structural differences including large chromosomal inversion and extensive variation in IS sequences regardless of the genetic similarity (30). The whole genome analysis of intermediately pathogenic *Leptospira licerasiae* demonstrates the proximity toward pathogenic than to saprophytic, also shedding a light into the genomic bases of infectiousness and its unique antigenic characteristics (31). Recently reported *Leptospira* genome has ∼1100 core genes with an open pan-genome suggests that entire gene repertoire is not yet expounded (32). In order to understand the genomic evolution and discriminate between pathogenic and non-pathogenic species including adaptation of pathogens to survive in critical environmental conditions, knowledge of GIs is crucial. Previous comparative genomic study revealed that *L. interrogans* serovar Lai strains 56 601 has four novel GI regions, which are associated with genes encoding transcriptional and virulence factors (33). The database also incorporates regions of GIs in plasmids along with chromosomal GIs for 16 strains with assembly status of complete genome. Evidently, our data suggested that more number of GIs are present in chromosome-I than chromosome-II for all the species with highest number of regions in *L. santarosai* serovars Shermani str. LT 821 amongst pathogenic strains. The serovar Linhai str. 56 609 is the only strain for which we found the GIs in its plasmid as well along with both the chromosomes. It has been found, that there is an extensive variation of gene count in all the analyzed strains. Most of the GI regions of pathogenic strains encompass hypothetical proteins indicating the probability of finding novel genes in turn implying in depth proteomic study for understanding the pathogenicity in case of *Leptospira.*

Lately, CRISPR/Cas has been recognized as a universal genome editing tool (34) and it is of vital importance to have knowledge and understanding of CRISPR/Cas9 systems for effective and specific targeting. The CRISPR/Cas9 systems of *Francisella novicida* a gram-negative bacterium, is itself involved in pathogenesis (35). Pioneering work by Marraffini and colleagues (2012) suggested that CRISPR/Cas9 system could be used for sequence specific killing (36). Recently reported, *L. interrogans* serovars Copenhageni strain Fiocruz L1–130 carries a set of Cas genes associated with CRISPR–Cas subtype I-B.

CRISPR-Cas system type-I is majorly found in *Leptospira* species as evident from our data along with the co-occurrence of type-III in *L. interrogans* serovar Bratislava strain PigK151, *L. interrogans* serovars Copenhageni str. Fiocruz L1-130 and *L. interrogans* serovars *Copenhageni str. FDAARGOS 203*. Fouts *et al*. have analyzed representative strains of Pathogenic, Intermediate and Saprophytic species and found CRISPR/cas in all the pathogenic strains, except *Leptospira borgpetersenii* and all saprophytic strains (37). While in our analysis, CRISPR/cas system was found in all serovars of *L. borgpetersenii* in addition to all the pathogenic serovars later being in congruence with their findings. So, our data coincide with the already available reports where CRISPR is found only in pathogenic members of the genus. Also, no CRISPR system was detected for saprophytic strains of *Leptospira* which also coincides with Fouts *et al*. findings*.* Chromosome-I of each *Leptospira* species contained CRISPR system whereas no CRISPR was found for chromosome-II and plasmid in accordance with the previous reports (37) which reflects the importance of Chromosome-I for further pathogenicity study. (i) As a whole with the advent of sequencing technology, the availability and visualization of data at a single platform to facilitate further analysis is significantly the need of the hour. As a result of phylogenetic analysis, the phylogenetic tree clearly segregated and formed different clades for various species. The three major clusters named clade A, clade D and clade B were obtained. The various serovars *Leptospira alexandri* (*n* = 6), *Leptospira alstonii* (*n* = 6), *L. borgpetersenii* (*n* = 37), *L. interrogans* (*n* = 341), *Leptospira kirschneri* (*n* = 29), *Leptospira kmetyi* (*n* = 1), *Leptospira mayottensis* (*n* = 2), *Leptospira noguchii* (*n* = 10), *L. santarosai* (*n* = 40), *Leptospira weilii* (*n* = 18) lie in clade A along with *Leptospira licerasiae*, *Leptospira wolfii*, *Leptospira broomi*, *Leptospira fainei* and *Leptospira inadai* which forms sub cluster designated as D. As evident from the tree, all the serovars in clade A have arisen from a common ancestor and have evolved parallelly. Few of the unclassified species of *Leptospira* (*n* = 7) also falls in the clade A exhibiting homology to pathogenic strains. As observed, clade B includes all saprophytic strains, which are *L. biflexa*, *Leptospira wolbachii*, *Leptospira vanthielli*, *Leptospira terpstrae*, *Leptospira yanagawae* and *Leptospira meyeri* separately forming a group exhibiting significant variation in sequence homology compared to serovars from clade A. *Leptonema ilini* and the clade C with *Leptospira parva* forms outgroup with separate clusters representing maximum variability compared to *Leptospira* species. Apparently, our analysis indicates that there is significant variation observed in the sequence homology of 16S rRNA gene reflecting genetic diversity among the various serovars of pathogenic strains in the first clade. Our data clearly clustered pathogenic, saprophytic and intermediate species into their respective groups. Therefore, our phylogenetic analysis may help in sequence comparison, identification and categorization of type of culture strains suspected to be of *Leptospira*. Additionally, signature sequences identified from this data may aid in designing primers suitable for *Leptospira* screening and identification.

### Diagnostics and therapeutics

The Global burden of leptospirosis in terms of mortality and morbidity demands availability of adequate diagnostics, therapeutics and preventive intervention strategies. The disease is usually diagnosed with microscopic agglutination test (MAT), which is a gold standard test but delays diagnosis while polymerase chain reaction (PCR)-based detection is sensitive and promising to rapidly confirm the diagnosis in the early phase of the disease. Our resource contains experimentally validated primer pairs based on gene specific target, most frequently used 16S, 23S and few based on genomic libraries for detection of both pathogenic and non-pathogenic Leptospires. The primers G1 and G2 are most widely used for clinical studies. The data availability at a public platform will aid future efforts to design primers for reliable diagnosis facilitating effective responses to potential epidemics.

Currently, it is indispensable and a challenge to develop drugs for Leptospirosis as very few structures of proteins are reported in PDB. Several discrete *in silico* studies have been done to find potential inhibitors against few putative targets like kdsA, LpxC (38) and GmhA, mur family proteins (39), which are involved in LPS biosynthetic pathway and peptidoglycan biosynthetic pathway, respectively. Our resource facilitates wide array of proteins ranging from different biochemical pathways including pathogen specific pathways to membrane and cytoplasmic proteins including enzymes and non-enzymes as putative targets, thereby attempting to bridge a gap in drug discovery for leptospirosis. Regardless of the steady progress in the field of development of anti-microbial therapeutics, vaccination strategies also needs to be improved to elicit effective method for people who constantly encounter infected animals and environment. For the development of effective and safe vaccine, knowledge on various proteins is also required like potential LPSs lipoproteins and proteins involved in bacterial motility. Currently, research on vaccine development against *Leptospira* is more concentrated on the discovery of cross-reactive conserved antigens that are able to elicit long-standing protection against a wide range of serovars of *Leptospira* species (40). There is a need to develop multi-epitope vaccine containing both T-cell and B-cell epitopes for effectiveness and better efficacy. The epitope data provided in database can be useful to the *Leptospira* scientific community for the design, characterization, and identification of potential vaccines and diagnostics, as well as to assist in basic investigation of immune responses and host–pathogen interactions. In conclusion, our database not only works as an informational database but also provides new biological insights. The information derived from our database and extensive future analysis may lead toward the development of possible molecular targets for this emerging disease and will indeed aid researchers and pharmaceutical agencies to conceive experiments for enriched development of vaccine and drugs against *Leptospira*. The present research group is also involved in extensive molecular and genetic analysis of various aspects of Leptospirosis toward potential therapeutics for this emerging disease. 

## Supplementary data

Supplementary data are available at *Database* online.

## Supplementary Material

Supplementary Data
