# Supplementary material for: LeptoDB: an integrated database of genomics and proteomics resource of Leptospira
Source: Database (Oxford). 2018 Jun 12;2018:bay057. doi: 10.1093/database/bay057 (PMC6007218; doi:10.1093/database/bay057)
Supplement: Supplementary Data [file Supplementary Figure S1.pdf › Supplementary Figure S1.pdf]

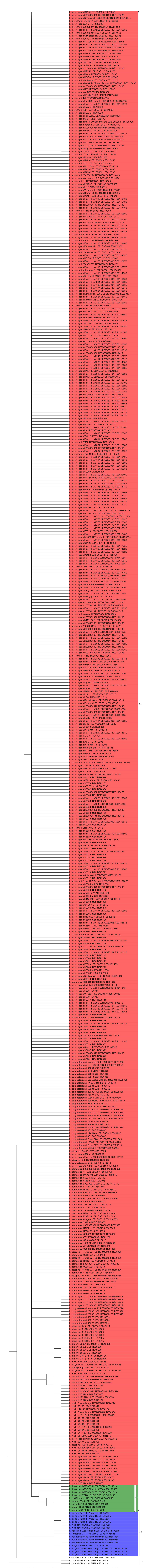

Supplementary Figure S1. 16sRNA based phylogeny tree of 524 strains(A-Pink color denotes pathogenic strains, D-Green color denotes intermediate strains, B-Purple color denotes saprophytic strains and C-strains with no color represents outgroup members)
